# Setting up a clinical trial for a novel disease: a case study of the Doxycycline for the Treatment of Nodding Syndrome Trial – challenges, enablers and lessons learned

**DOI:** 10.1080/16549716.2018.1431362

**Published:** 2018-01-31

**Authors:** Ronald Anguzu, Pamela R Akun, Rodney Ogwang, Abdul Rahman Shour, Rogers Sekibira, Albert Ningwa, Phellister Nakamya, Catherine Abbo, Amos D Mwaka, Bernard Opar, Richard Idro

**Affiliations:** ^a^ Department of Paediatrics and Child Health, Makerere University College of Health Sciences, Uganda; ^b^ Department of Paediatrics and Child Health, Centre of Tropical Neuroscience, Kitgum Site, Uganda; ^c^ Institute of Health and Equity, MoH, Medical College of Wisconsin, Kampala, USA; ^d^ Ministry of Health, Headquarters, Uganda; ^e^ Nuffield Department of Medicine, University of Oxford, Oxford, UK

**Keywords:** Nodding syndrome, randomized clinical trial, doxycycline, Kitgum General Hospital

## Abstract

A large amount of preparation goes into setting up trials. Different challenges and lessons are experienced. Our trial, testing a treatment for nodding syndrome, an acquired neurological disorder of unknown cause affecting thousands of children in Eastern Africa, provides a unique case study. As part of a study to determine the aetiology, understand pathogenesis and develop specific treatment, we set up a clinical trial in a remote district hospital in Uganda. This paper describes our experiences and documents supportive structures (enablers), challenges faced and lessons learned during set-up of the trial. Protocol development started in September 2015 with phased recruitment of a critical study team. The team spent 12 months preparing trial documents, procurement and training on procedures. Potential recruitment sites were pre-visited, and district and local leaders met as key stakeholders. Key enablers were supportive local leadership and investment by the district and Ministry of Health. The main challenges were community fears about nodding syndrome, adverse experiences of the community during previous research and political involvement. Other challenges included the number and delays in protocol approvals and lengthy procurement processes. This hard-to-reach area has frequent power and Internet fluctuations, which may affect cold chains for study samples, communication and data management. These concerns decreased with a pilot community engagement programme. Experiences and lessons learnt can reduce the duration of processes involved in trial-site set-up. A programme of community engagement and local leader involvement may be key to the success of a trial and in reducing community opposition towards participation in research.

## Background

Randomized clinical trials (RCTs) are considered the criterion standard to assess the efficacy of interventions or treatment [1]. Conducting RCTs is time-consuming and expensive [2,3]. Complexity of trial conduct usually occurs in multiple sites or those focused on poorly understood disorders or in emergencies. Physical access issues such as distant sites and community perceptions further complicate site set-up. Trials may experience logistical challenges such as sophisticated equipment and materials, which may be costly or unavailable [4].

Experiences from the increasing number of clinical trials conducted in low-income countries is seldom reported [5,6]. The complexity of implementing trials includes lengthy procedures for study approval, regulatory processes and community engagement needs, which make trial set-up labour-intensive and costly [7]. In addition, the process of testing Investigational Medicinal Products and material procurement such as equipment or drugs often depends on intra- and inter-institutional factors, which may be protracted and challenging [8]. In Uganda, most clinical trials have focused on infectious diseases such as malaria, HIV/AIDS and tuberculosis. Infrastructural demands, scarcity of trained personnel and lack of funding are also common limitations. Other hindrances to setting up trials such as site identification, methodological concerns related to study design, ethics and results interpretation with implications for practice and policy are poorly documented.

Planning for clinical trial site initiation typically begins before protocol development with the conduct of feasibility assessments for their suitability and readiness in respective settings [9]. As part of the planning process, investigators need to consider building a qualified implementation team, assessing the local environment and complex dynamics of the target community, infrastructure needs and the potential to conduct and complete the trial within the proposed timelines. The process of involving communities in the research process is becoming increasingly important because this demonstrates mutual respect for participants [10]. From a community perceptive, engagement of the general public enables their understanding of ethical concerns such as a participant vulnerability [11].

Nodding syndrome is a devastating neurological disorder of unknown cause affecting children in Eastern Africa [12]. Head nodding is the pathognomonic symptom with onset in children aged 3 to 18 years [13]. Subsequent complications include multiple seizure types, cognitive decline, behavioural problems, psychiatric disorders, severe physical disability, malnutrition, and delayed physical growth and sexual development [14]. Symptoms, however, improve with symptomatic treatment [15]. Many deaths have been reported often associated with status epilepticus, drowning and severe burns [12]. To date, the only strong aetiologic association is infection by *Onchocerca volvulus* [16]. More recent pilot studies suggest that nodding syndrome may be a neuro-inflammatory disorder with antibodies to *O. volvulus* cross-reacting with host neuron proteins [12,17]. Our study, *Doxycycline for the Treatment of Nodding Syndrome* (ClinicalTrials.gov NCT02850913), is a novel trial [18] with a concurrent nested case–control study investigating the cause and pathogenesis of nodding syndrome. The trial is examining the efficacy and safety of 100 mg of oral doxycycline or placebo daily for six weeks as treatment for nodding syndrome in Uganda. This paper describes experiences and documents the supportive structures (enablers), challenges faced and lessons learnt during the set-up of the trial.

## Methods

### Design

This is a case study for the set-up of the ‘Doxycycline for the Treatment of Nodding Syndrome’ trial in Kitgum General Hospital. The trial is a phase II randomized placebo-controlled study of oral doxycycline 100 mg or placebo daily for six weeks. The objective is to determine if nodding syndrome is a neuro-inflammatory disorder induced by *O. volvulus* or its co-symbiotic bacteria, *Wolbachia*, and whether the intervention can improve outcomes. Recruitment of all 230 participants is expected to last approximately 15 months and subsequently followed up for another 24 months.

The trial will hospitalize eligible participants for 1–2 weeks in order to conduct baseline tests, namely clinical, electroencephalography, cognitive and laboratory assessments, and rationalization of anti-epileptic drug doses. The trial intervention (doxycycline or placebo) will be initiated in Kitgum General Hospital (KGH) prior to discharge. Each participant will thereafter have scheduled visits in their homes in the 2nd, 4th and 6th weeks for adherence and safety monitoring. Similar scheduled follow-up visits will be conducted in the 3rd, 6th, 12th and 18th month visits in peripheral clinics or KGH. Unscheduled visits, i.e. assessments occurring between specified follow-up visits for unanticipated illnesses, are expected. At 24 months after the intervention, all participants will be assessed for the primary outcome (end-point) in the KGH. The trial primary outcome will be the proportion of patients with antibodies to neuron surface proteins (leiomodin) in the 24th month.

This case study describes the step-by-step activities involved in setting up this clinical trial and preparations made to operationalize the trial site.

### Setting

The study area includes the districts of Kitgum, Pader and Lamwo in Northern Uganda with a 2016 mid-year population projection of 209,600, 183,500 and 137,000 respectively [19]. This community is predominantly involved in agricultural activity for subsistence inhabited mainly by people belonging to the Acholi ethnic group. The population prevalence of nodding syndrome in the affected region is 6.8 (95% CI 5.9–7.7) per 1000 [20]. The region has high poverty levels, psycho-socio problems and neglected tropical diseases, and until recently, most of the population lived in internally displaced people’s camps during a protracted two-decade civil war. KGH is a public health facility and hosts the main nodding syndrome referral centre in addition to hosting our clinical trial centre. Other treatment centres are smaller health centres across the northern region of Uganda [12].

### Approach and processes

From September 2015 to 5 September 2016, the study team conducted pre-site initiation activities, namely: design and submission of the trial protocol for ethical approval, developing essential trial documentation, engaging community and district leadership, conducting pre-visits to nodding syndrome treatment sites, recruiting and training trial staff, and procurement of study-related items.

#### Discussions with village and district leaders

Stakeholder meetings were held with community, district and political leaders and their views and opinions documented in the proceedings. Family invitations were issued during home visits with field teams to participate in community engagement meetings. These attendances included random groups of both women and men aimed at introducing the proposed trial, encouraging platforms for open dialogue inclusive of women’s voices being heard and taken account of. During community dialogue meetings, village leaders and other attending residents were predominantly male compared with females who are primarily caretakers of children.

Discussions with the district and hospital leaders aimed to introduce the trial, seek collaboration and advocate for support for the study. Experiences of the study team members during site set-up and trial preparation were also described in our field activity reports.

#### Site pre-visits in nodding syndrome treatment centres

Initial pre-visits were conducted in three nodding syndrome treatment centres in order to assess their feasibility for participant screening, recruitment and conduct of study-specific tests. Medical records in treatment centres and nodding syndrome registers held by VHTs with nodding syndrome and epilepsy patients was reviewed to estimate their respective proportions and sampling frame for recruitment.

#### Feasibility assessment of the trial centre

The physical infrastructures in the peripheral nodding syndrome treatment sites and hospital trial centre were assessed for suitability. The trial team evaluated the units’ adequacy of working space to conduct study procedures, availability and reliability of electricity, and Internet connectivity for effective communication. We also evaluated the proposed study laboratory for cold chain maintenance capacity and assessed available transportation options for the study participants, samples and team.

#### Development and design of protocol and essential trial documents

Trial protocol development started in September 2015 followed by a 6-month consultative and training process by study investigators to engage trial staff and other stakeholders for the development of essential trial documents. Brainstorming sessions with stakeholders were conducted to develop draft and final versions of standard operating procedures and case report forms (CRFs), and described in pre-visit activity reports.

#### Recruitment and training of the trial team

Eight core study team members were recruited in a phased approach. This was followed by recruitment of five hospital-based laboratory and nursing locum-based staff who underwent a five-day training course on Good Clinical Practice and Human Subjects Protection. Selected trial staff also underwent training on specialized study procedures and assessment tests such as electroencephalography and cognitive assessments using CogState®, and reviewed the CRFs.

#### Protocol submissions for ethical review committee approval

For ethical approval, study protocols were submitted to Makerere University School of Medicine Research Ethics Committee (SOMREC), Uganda National Council of Science, and Technology (UNCST) and University of Oxford Tropical Research Ethics Committee (OxTREC). The consent forms were provided for permission to collect, store and transport samples outside Uganda for further antibody, genetic and biomarker testing. Material Transfer Agreements were sought from UNCST to transport human subject samples for immunologic, molecular biology and genetic testing in the University of Oxford Immunology, University College of London – Institute of Neurology lab, the Wellcome Trust Genetics Centre at Sanger in UK and the Kenya Medical Research Institute – Wellcome Trust Research Collaboration in Kilifi, Kenya. The protocol was also submitted for regulatory approval to the National Drug Authority (NDA) and was registered with clinicaltrials.gov [18].

### Conduct of administrative processes

Institutional systems of Makerere University, Kitgum District Local Government and KGH were utilized to facilitate phased recruitment of the implementation team, procurement, staff payment and refurbishment of the trial building.

## Analysis

Observations by the implementation team and proceedings of meetings with the village health teams, clinicians and community leaders were summarized as statements. The frequency and mean duration (in months) from submission to receipt of protocol-related items were calculated using MS Excel 2013.

## Results

We describe the challenges, supportive structures (enablers) and lessons learnt during the trial set-up of a clinical trial site in the nodding-syndrome-affected districts of Northern Uganda.

## Challenges experienced

### Systemic community issues

The community involved in this study has one of the highest poverty levels in Uganda. This was worsened by the post-war sequelae of psychological and social problems such as orphan hood, child-headed households with a high dependant population and food insecurity leading to dependence on food aid. This trial was often perceived as a food-relief effort and not as a new health intervention whose intention is mainly to identify the cause and treatment of nodding syndrome.

### Community leader and other stakeholder perceptions and concerns

Multiple myths and suspicions existed that could potentially hinder community entry for the conduct of research. At both district and community level, the major concerns were: (1) minimal or no public dissemination of previous research findings on nodding syndrome to affected communities; (2) lack of an effective community engagement strategy; and (3) psycho-socio-economic issues. Some suggestions from stakeholders underpinned the importance of involving the community during trial set-up and the need to put in place strategies to ensure consistency in feedback channels of study progress at trial onset, interim and closure.

### Negative community concerns towards scientists and research on nodding syndrome

Concerns from community members, village health teams and clinicians pre-visited were associated with anxiety about the cause of nodding syndrome, severity of the disease and the devastation it is continuing to cause, long period of time taken to identify the cause and poor feedback to the affected communities about research progress to date. There was community mistrust and ‘fatigue’ towards research and scientists. Communities felt that authorities and scientists were deliberately withholding information on research findings. Community fatigue and frustrations should be systematically addressed in order to reduce resistance.

#### Poor physical access to nodding syndrome treatment centres

**Figure 1.**
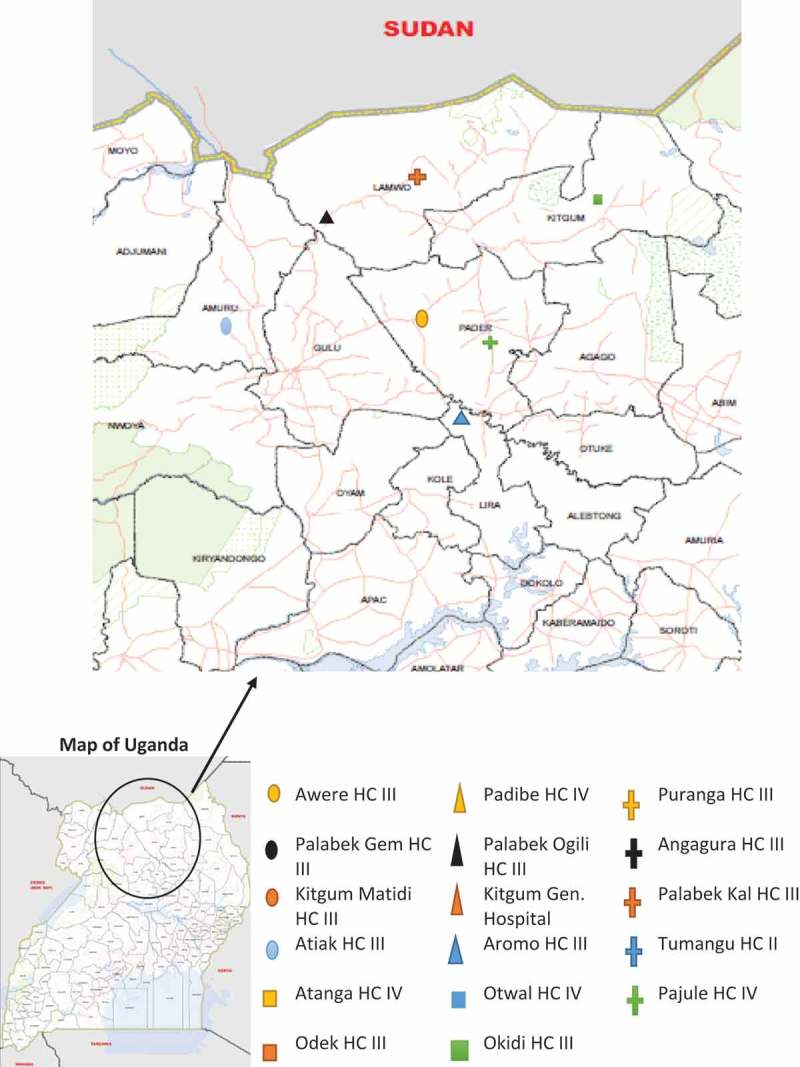
Duration to completion of pre-trial initiation activities and receipt of protocol related submissions, 2016.

The first three nodding syndrome treatment centres pre-visited, i.e. Kitgum Matidi HCIII, Okidi HCIII and Tumangu HCII (Figure 1), were located in hard-to-reach settings; yet they attended to high numbers of patients with nodding syndrome and other forms of epilepsy. This study requires consistency in maintenance of participant samples under optimal conditions for storage and transportation. Frequent long distances are expected to be travelled by trial staff and potential participants. This barrier posed a potential risk for disruption of the cold chain for study samples. Monitoring trial drug adherence would also be potentially physically challenging in light of multiple home visits expected to be conducted.

**Figure 2.**
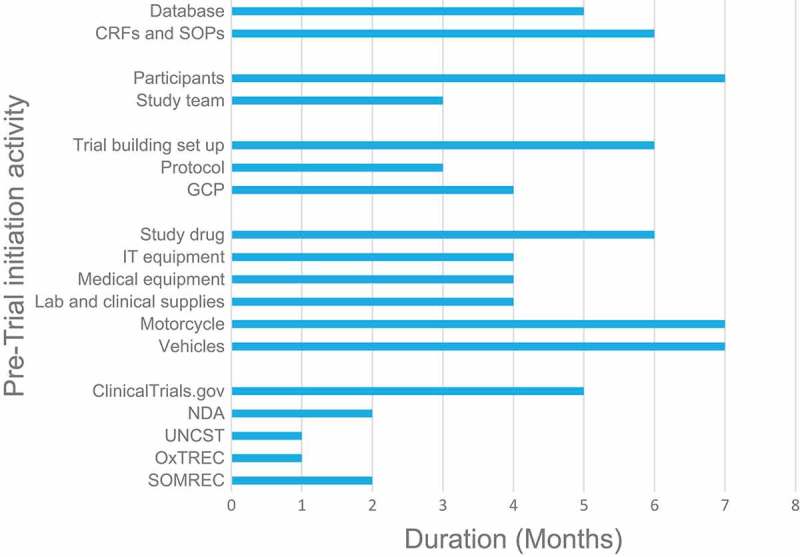
Map showing the clinical trial site in Northern Uganda.

#### Prolonged duration for receipt of ethical review approvals

The time taken between submission of the trial protocol and receipt of ethical approval was lengthy. In Figure 2, obtaining the five required study approvals took an average of 2.8 months (SD = 1.33). In addition, receipt of other protocol-related items such as supply and equipment procurement took a long time from submission [5.2 months (SD = 1.47)].

#### Impairment of cognition in nodding syndrome

Patients with nodding syndrome often present with cognition impairment, and so most participants will potentially have diminished capacity to provide consent or assent. Considerations were made during the trial design for development of caregiver consent and participant assent forms. This is because most patients with nodding syndrome are expected to have diminished autonomy for informed decision-making owing to impaired cognition.

### Supportive structures

#### Availability of nodding-syndrome-specific trained healthcare workers in the treatment centres

The three pre-visited nodding syndrome centres had healthcare workers trained in the clinical management and care of patients with nodding syndrome and other epilepsies. Unscheduled visits to these centres are expected, and so the trial staff further trained these clinicians on study procedures, their role in treatment and administration of concomitant medications including avoidance of prohibited drugs during the 24-month follow-up period.

#### Village health teams (VHTs)

The VHTs were enlisted to support the trial recruitment process. These community health workers are available in each village, and most have received training in the community surveillance for nodding syndrome cases. Peer VHT supervisors have maintained updated registers of patients with confirmed nodding syndrome and epilepsy in their villages since the initial outbreak-response efforts in 2012. The VHTs also support participant follow-up in the community following discharge after treatment initiation from the main trial centre. In the trial, participant follow-up in the villages will be conducted at 2, 4 and 6 weeks and at 3, 6, 12, 18 and 24 months when the primary end-point is determined. The VHTs will also play a role in improving (1) continuation rates, (2) early identification of adverse events and (3) dissemination of information on our research objectives, risks and benefits. Community mobilization is expected to be conducted within respondents’ homes and health facilities, and through public dialogue within the catchment area of selected VHTs.

#### Support and investment from KGH, central and local government

We received both administrative and political support from Kitgum district local government and Uganda’s Ministry of Health. Infrastructure in the form of a building was donated to the trial, and we refurbished it into a study office in order to support hospitalized participants during inpatient clinical observations and monitoring. The host hospital also committed support to the trial in the form of health workers in the nodding syndrome ward to provide clinical care including the management of serious adverse events. Laboratory-certified technicians and adequate lab space with capacity to conduct study specific tests and to store blood and cerebro-spinal fluid (CSF) samples were also offered. Although rather slow, the hospital’s state-funded Internet connectivity was made available, and this will be leveraged for timely communication. In the trial design, we anticipated partial electronic data collection for which a secure and reliable Internet and back-up system was critical.

In-depth discussions were held with individual staff to guide development of their research ideas and pre-doctoral study discussions as part of the clinical trial’s academic capacity-building component for the implementation team.

#### Site initiation monitoring visit

Our core and support trial members were involved in training on study procedures and Good Clinical Practice (GCP). Independent trial monitors conducted two days’ training on GCP and Human Subjects Protection (HSP). This was intended to improve staff readiness to start participant recruitment while maintaining good ethical conduct and data quality. Emphasis was made on collecting quality data by maintaining consistency, correctness and completeness.

## Lessons learned

Setting up a clinical trial in hard-to-reach areas can be a protracted process owing to multiple logistical, infrastructural, trial and administrative requirements. Potential solutions to the often very slow and multi-level processes for procurement and other trial-related activities need to be explored or identified. Strategies to re-introduce the research agenda for nodding syndrome in Northern Uganda will require involvement of the community in the research-planning process. Potential needs exist to: (1) provide information about research and progress (dissemination), (2) consult with members and leaders about their views (dialogue) and (3) collaborate with members and scientists to plan, implement and propose recommendations (partnerships).

## Discussion

This paper describes the challenges experienced, supportive structures (or enablers) and lessons learnt during the process of setting up a trial site for nodding syndrome research in the affected districts of Northern Uganda. To the best of our knowledge, we provide the first findings describing experiences of setting up this trial site for nodding syndrome research. The lessons learned may be benchmarked by similar trials in future.

Our feasibility assessments of the main and peripheral health for staff and infrastructural availability showed readiness for trial site activation. As recommended by the International Council on Harmonisation, our trial staff were trained in trial conduct with trainings in GCP and HSP [21] fulfilling this key criterion. Our trial was further enabled by the availability of appropriate key infrastructure such as a certified laboratory, a refurbished study building and Internet access. Scaling up infrastructural and financial support has been shown to reduce delays to trial site activation and is critical for the conduct of high-quality clinical trials [22].

In our clinical trial, we anticipate a complex, labour-intensive and costly implementation where participants will be clinically assessed in the hospital trial unit, peripheral health centres and home visits. These follow-ups or scheduled assessments in the 24 month follow-up period will be utilized to reinforce risks and benefits of volunteering to participate and manage any adverse events, study drug and anti-epileptic medication and clinical assessments as required per protocol [18].

Views from health workers and VHTs suggest a need for involvement of the general public in order to address community mistrust and misperceptions. These perceptions are suggestive of a societal need to dispel misunderstandings about nodding syndrome studies that could negatively affect participant recruitment. Reversing these undesirable views of communities towards research can be addressed by increasing community literacy of research. Community awareness of ongoing or new clinical trials increases willingness for research participation, accessibility to research information and ‘visibility’ by the general public [23]. Evidence suggests that community entry for research through public dialogues can be leveraged to explain details of the interventions in order to improve community literacy about research, thereby dispelling misconceptions. Our trial is expected to use public dialogue as a communication channel to disseminate information about our study and in turn provide answers to some community concerns.

In our study area, most community leaders are men, and most decisions including healthcare seeking decisions are made by men. Conversely, the burden of caretakers of children with nodding syndrome is socially skewed to the women [24]. However, the challenges of caregiving of children with nodding syndrome may not be well understood by men [25]. We believe that community engagement including both men and women is a reasonable approach to recruitment and commitment to the trial.

A systematic review by Bonevski *et al*. identified different categories of socially disadvantaged groups such as women of low-income status for targeted strategies to increase their involvement in health research [26]. One study contested the view that although recruitment of women into clinical trials may present formidable challenges, follow-up rates may not be associated with their low-income or minority status [27]. Underserved populations in this hard-to-reach trial site potentially have poor geographical access to health services and research. This often results in little understanding of their poor health outcomes [28]. In addition, a related study highlighted a lack of transport, healthcare costs and literacy level as barriers to trial recruitment [29]. Building culturally competent approaches and trusted community–researcher relationships is important for improvement of research recruitment and retention among socially vulnerable groups such as women or youth [30].

Clinical trials involving idiopathic medical conditions may raise ethical issues, especially if an individual’s autonomy for decision-making is impaired as in nodding syndrome. Elsewhere, experiences from community engagement suggest that taking into consideration the influence of the wider community on individual consenting, potential risks and benefits that community perspectives may have is important [31]. In addition, GCP demands that ethical issues among both the trial participants and the affected communities be addressed [32]. A review of studies in low- and middle-income countries showed most to have near universal community support for the implementation of interventions. However, one-fifth of studies did not involve the community in participating in identifying or defining problems and community members for participation [33]. In coastal Kenya, community engagement activities using local residents addressed community myths towards biomedical research [34,35]. Involving the general public in the planning process can increase their willingness to participate in research and reduce loss to follow-up [23]. In our trial, home visits are planned every fortnight for six weeks in addition to scheduled three- and six-monthly visits up to the 24-month end-point. This follow-up approach at household and health facility level is expected to improve continuation rates, adherence to both anti-epileptic and study drugs, and identification of adverse events.

New National Institutes of Health recommendations suggest that incorporation of sex as a biological variable is valuable and can impact pre-clinical neuroscience research [36–38]. Regarding the trial, inclusion of sex or gender was factored in the protocol design and follow-up process in order to mitigate barriers to recruitment and follow-up. In our study, for example, at the trial design level, randomization to eliminate selection bias owing to individual characteristics such as sex was conducted. In addition, pregnant women will be excluded owing to the potential risk of congenital abnormalities by the trial drug, and pregnancy tests were also conducted every 2 weeks among female participants to further reduce this risk. At intervention and follow-up phases, women as primary caregivers will receive physical and emotional support for care provision during the trial pre- and post-hospitalization phases for potential long-term improvement of their quality of life. Our study findings were similar to evidence elsewhere suggesting that increased involvement and inclusion of minority populations in health research design and implementation contribute to a reduction in negative perceptions towards research and increased participation [39]. In a separate paper among a series from the trial being drafted by the Centre for Tropical Neurosciences in Uganda, the authors will detail the process of developing and implementing a community engagement strategy and further describe the role of women in improving trial participation.

Several ethical and regulatory approvals were required. Elsewhere, such delays are underpinned as reasons for the prolonged duration to start recruitment. Our experience concurred with a trial that obtained individual approvals within three months [40]. Similar formal administrative steps prior to approval were undertaken in the previous trial. Owing to different requirements, it can be assumed that multi-site trials may experience longer periods to receive ethical approval compared with single site trials such as ours. In a multi-centre trial and a retrospective feasibility assessment of multiple trials, it took twice as long for ethical approvals to be obtained compared with our trial [8,41]. Common reasons for delays were unsuitability of the treatment setting and pharmaceutical delays. These delays were noted to be major barriers to the conduct of health research highlighting a need to simplify processes involving multi-centre trials, having fixed timelines targeting pre-trial feasibility assessments and receipt of approval.

A systematic review of trial site performance showed longer delays to trial sites opening in high-income countries (median time = 250 days; 188–266) [9] than in our trial. Our study was, however, multi-jurisdictional requiring different ethical approvals and one regulatory obligation [42–44]. Common reasons for delays to receipt of approvals were mainly late submission of IRB responses and, from regulatory authorities, included: multiple reviewer queries and issues regarding use of the study drug for the treatment of nodding syndrome.

We also experienced delays in procuring equipment and supplies for as long as 6 months. Previous trials cited a lack of legal units to deal with contracts as a reason for logistical delays [8]. Delays during our trial set-up could be due to multiple requirements in the procurement cycle. In a review of Phase III trials, process mapping of activities could identify pre-trial initiation challenges such as multiple steps or decision points prior to participant recruitment [45]. It should be noted that not all findings from this case report may be transferable to experiences of setting up trials in all contexts. Reporting bias could also have arisen owing to transcriptions of discussions verbatim.

## Conclusion

Our experiences and the lessons learned in this trial set-up may help reduce the duration of processes involved in trial site set-up. This set-up may provide a benchmark for the conduct of similar trials and trials involving similar conditions or in similar settings in future. A programme of community engagement and local leader involvement that is culturally competent and gender-sensitive may be key to success and in reducing community opposition towards participation in research. Formation of community advisory boards and engagement teams is recommended.

## Paper context

Nodding syndrome is an acquired neurological disorder of childhood onset in Sub-Saharan Africa with no known cause and treatment to date. Our paper describes our experiences in preparing this hard-to-reach site for a randomized controlled trial testing the first potential treatment for nodding syndrome and a nested case–control study to identify its cause. Engagement of local leaders and dialogue with residents in hard-to-reach areas addresses community anxiety and willingness to participate in the trial.
